# Supplementary material for: Discovery, Pharmacological Characterisation and NMR Structure of the Novel µ-Conotoxin SxIIIC, a Potent and Irreversible NaV Channel Inhibitor
Source: Biomedicines. 2020 Oct 2;8(10):391. doi: 10.3390/biomedicines8100391 (PMC7599555; doi:10.3390/biomedicines8100391)

## Supplementary Material

# Discovery, pharmacological characterisation and NMR structure of the novel μ-conotoxin SxIIIC, a potent and irreversible Nav channel inhibitor

**Kirsten L. McMahon [1], Hue N.T. Tran [1], Jennifer R. Deuis [1], Richard J. Lewis [1], Irina Vetter [1,2,*] and Christina I. Schroeder [1,3,*]**

1 Institute for Molecular Bioscience, The University of Queensland, Brisbane, QLD 4072, Australia;
2 The School of Pharmacy, The University of Queensland, Woolloongabba, QLD 4102, Australia;
3 National Cancer Institute, National Institutes of Health, Frederick, MD 21702, USA
* Correspondence: i.vetter@uq.edu.au (I.V.); christina.schroeder@nih.gov (C.I.S.)

**Figure S1: Activity of SxIIIC across Nav channel subtypes assessed by whole-cell patch-clamp electrophysiology.** Time course (current amplitude vs time) of SxIIIC at each subtype tested. Arrows indicate peptide addition at increasing concentrations following a 5 min buffer incubation. Currents were normalized to the buffer (0–100 s).

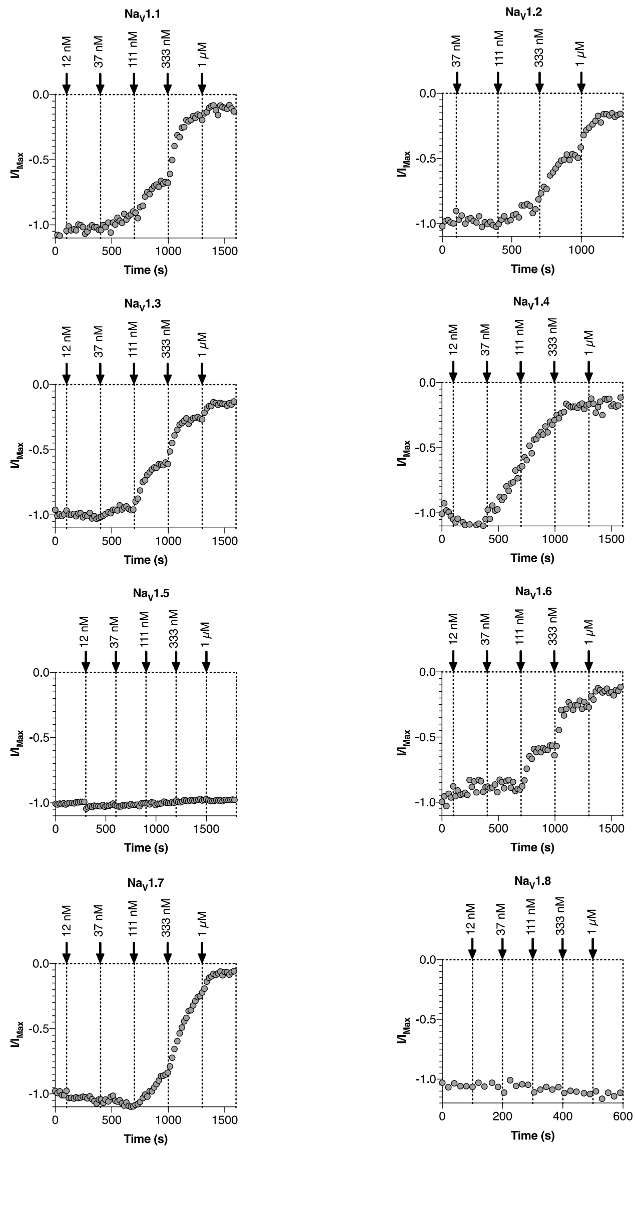

**Figure S2: SxIIIC superimposed over the KIIIA/Na$_V$1.2 structure.** SxIIIC is represented by grey/purple surface, while the Na$_V$1.2 channel is represented by ribbons with Domains I, II and III shown in blue, green and yellow, respectively. Insert shows a top view of this interaction in greater detail; residues from extracellular loop of Domain I (black labels) appear to clash with residues found on loop 1 of SxIIIC (white labels). The figure was generated in PyMol using the inbuilt function "super" to superimpose SxIIIC over KIIIA, in the KIIIA/Na$_V$1.2 structure (PDB ID: 6J8E).

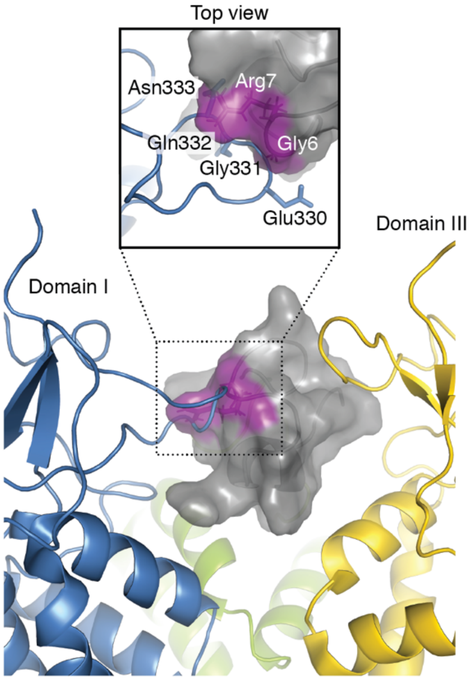

Supplement: Supplementary file 1 [file biomedicines-08-00391-s001.pdf]
